# Head orientation and electrode placement potentially influence fetal scalp ECG waveform

**DOI:** 10.1371/journal.pone.0223282

**Published:** 2019-10-10

**Authors:** Alexandra D. J. Hulsenboom, Guy J. J. Warmerdam, Janna Weijers, Paul J. Blijham, S. Guid Oei, Judith O. E. H. van Laar, Rik Vullings, Tammo Delhaas

**Affiliations:** 1 Department of Obstetrics and Gynaecology, Máxima Medical Center, Veldhoven, the Netherlands; 2 Faculty of Electrical Engineering, Eindhoven University of Technology, Eindhoven, the Netherlands; 3 Department of Clinical Neurophysiology, Máxima Medical Center, Veldhoven, the Netherlands; 4 Department of BioMedical Engineering, Maastricht University Medical Center, Maastricht, the Netherlands; Antwerp University Hospital/University of Antwerp, BELGIUM

## Abstract

**Background:**

Fetal monitoring based on electrocardiographic (ECG) morphology is obtained from a single unipolar fetal scalp electrode. Ideally, it should be obtained from multiple leads, as ECG waveform depends on alignment between electrode and electrical heart axis. This alignment is unknown in fetuses. Besides, fetuses are surrounded by conductive media, which may influence ECG waveform. We explored the influence of electrode position and head orientation on ECG waveforms of unipolar and bipolar scalp ECGs recorded in air and in conductive medium.

**Methods:**

We recorded ECGs in one adult subject at five different scalp positions in five different head orientations both in dry and immersed conditions. The ratio between T-amplitude and QRS-amplitude (T/QRS ratio) of unipolar and bipolar scalp ECGs was determined and compared between all conditions.

**Results:**

In the dry condition, we observed in the unipolar leads little to no difference between different electrode positions (maximal T/QRS difference 0.00–0.01) and minor differences between head orientations (0.02–0.03), whereas bipolar leads showed no recognizable ECG signal at all. During the immersed condition, we found variation in the unipolar leads, both between electrode positions (maximal T/QRS difference 0.02–0.05) and between head orientations (0.03–0.06). Bipolar leads showed different ECG signals in contrasting head orientations.

**Conclusions:**

Both unipolar and bipolar scalp lead-derived ECG waveforms are influenced by electrode position and head orientation when the subject is submerged in a conductive medium. Fetal monitoring based on single scalp lead ECG waveform might be suboptimal, as it lacks correction for fetal head orientation and electrode position.

## Introduction

Monitoring of the fetal condition during labor remains a great challenge in obstetric care. Information about the fetal condition can be obtained from the fetal electrocardiogram (ECG) because it provides both fetal heart rate and ECG morphology. The diagnostic value of the ECG morphology analysis to monitor the fetal condition was first demonstrated in animal studies, in which it was shown that the ST-segment changes under influence of oxygen deficiency.[1,2] Nowadays, ST–analysis (STAN®, Neoventa Medical AB, Mölndal, Sweden) is available in clinical practice, where the ECG is measured from a single unipolar fetal scalp electrode.[3]

Combined information from multiple leads is essential to correctly interpret ECG waveform. Amplitudes of the ECG waveform differ between different leads, since the alignment of the different electrodes and the electrical heart axis differs. During fetal ECG measurement in labor, only a single unipolar lead is used. Correct interpretation of the waveform is difficult, as the orientation to the electrical heart axis is unknown.[4] Besides, the fetus is surrounded by conductive media such as amniotic fluid and maternal tissue. We hypothesize that these conductive media influence ECG waveform and thereby impair correct interpretation of the signal.

In this study, we examined our hypothesis in an experimental setting. We examined the influence of electrode position and head orientation on the waveform of unipolar and bipolar scalp ECGs when recorded in air or in a conductive medium.

## Materials and methods

### Study population

In this experimental case-study we included one healthy member of our research team after informed consent. The ECG was recorded from various electrodes positioned on the scalp during different head orientations, both in a dry condition and an immersed condition. The Daily Board of the Medical Ethics Committee Máxima Medisch Centrum reviewed and approved the research proposal. The data, analytic methods, and study materials will be made available to other researchers for purposes of reproducing the results or replicating the procedure. The data that support the findings of this study are available from the corresponding author upon reasonable request.

### Materials and experimental set-up

**Fig 1:**
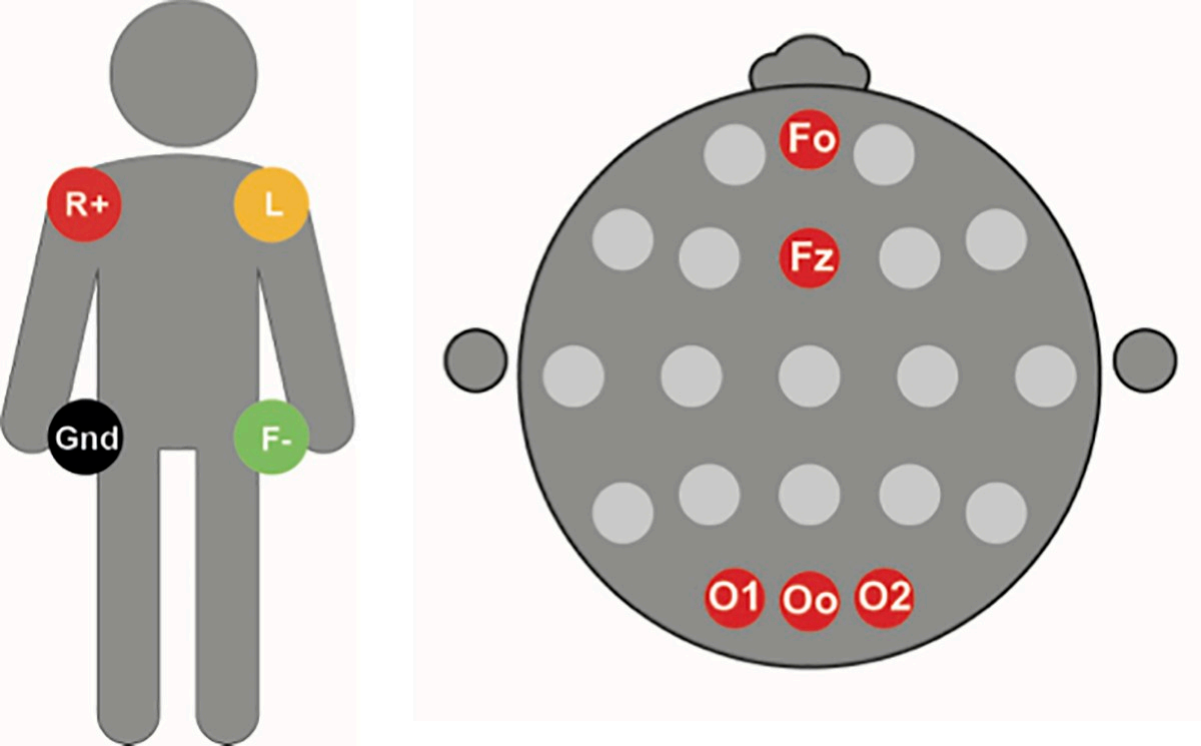
Schematic representation of electrode positions. Left panel depicts electrode positions on the rump. R, Right Arm; L, Left Arm; F, Foot; Gnd, Ground. Right panel represents electrode positions on the head in the 10/20 system. Positions F0, Fz, O1, Oo and O2 were used (marked in red).

Nine gold cup electrodes were placed on the participant’s skin: two at each shoulder, two at each superior anterior ischiadic spine, and at five positions of the 10/20 system: on the midline between Fp1 and Fp2 (named F0), on Fz, O1, O2, and on the midline between O1 and O2 (named O0) (Fig 1). Fig 1 represents the configuration of the electrodes. An experienced clinical neurophysiology technician placed the cup electrodes with Elefix as an insulator, Ten20 as conductive paste, and colloid for optimal fixation. We measured the lead of the scalp ECG as the difference between one electrode at the scalp and the Wilson Central Terminal (WCT), as recorded from the conventional ECG configuration at the rump.[5] In addition, we measured bipolar leads, by calculating the potential difference between pairs of electrodes on the scalp.

**Fig 2:**
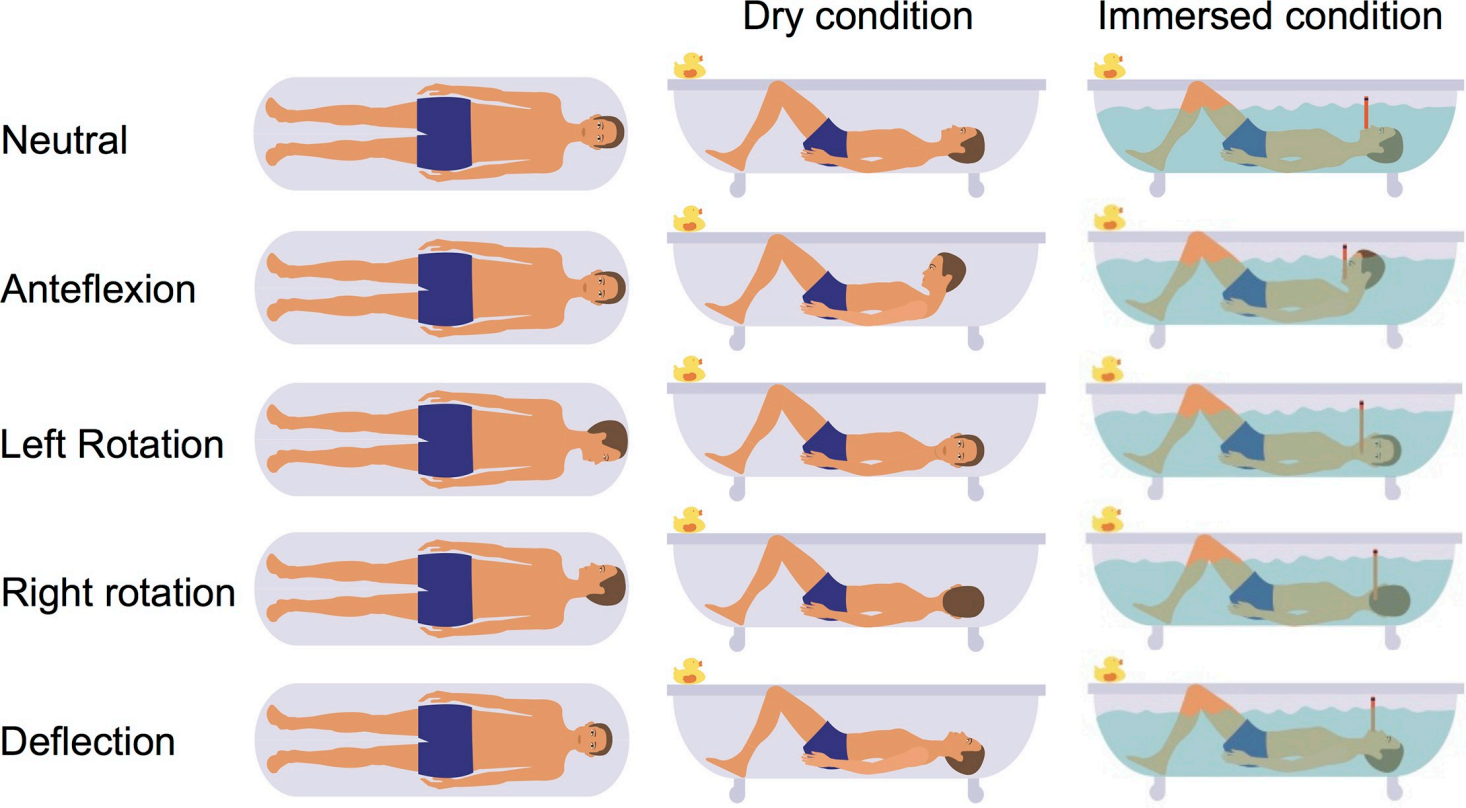
Body orientations. The panel shows five body orientations from top view, side view in dry condition, and side view in immersed condition, consecutively.

For the immersed condition, a saline solution at 37 degrees Celsius was used to simulate similar conductive conditions as the amniotic fluid (electrical conductivity of 1.6*10^−4^ Ohm^-1^cm^-1^).[6] The subject was lying in a bathtub in supine position and breathing through a snorkel. The ECG was measured in two consecutive sessions. First in dry, then in immersed condition. During both sessions, the same protocol of head orientations was followed: head neutral, anteflexion, right rotation, left rotation and deflexion. Fig 2 depicts the body orientations. Each head orientation was maintained for 20 seconds, with the subject holding its breath after exhalation. The experiment was conducted twice to evaluate reproducibility.

### Data-processing

The electrodes were connected to a commercial amplifier for electrophysiological measurements (Porti, TMsi, the Netherlands). All data were stored and processed offline. Data from the first and final 2 seconds during a head orientation were discarded to exclude potentially poor quality signals during the transition from one head orientation to the next. The signals were pre-processed by applying a bandpass filter between 2-150Hz to minimize the effects of low frequency baseline drift and high frequency noise. A 50-Hz notch filter was used to suppress the powerline interference.

After pre-processing, one average ECG complex was calculated for each electrode position in each head orientation in both dry and immersed condition as a unipolar and a bipolar lead. At first, the R-peaks were detected with a wavelet-based R-peak detection algorithm.[7] Subsequently, individual ECG complexes were aligned on their R-peak locations and an initial average ECG complex was calculated from these aligned ECG complexes. Then, each individual ECG complex was compared to this average ECG complex. Individual ECG complexes with a correlation of less than 0.9 with the average ECG complex were excluded for further analysis. The average ECG complex was re-calculated using only the included ECG complexes. Finally, based on the average ECG complex, the ST-segment was detected as the segment from S-peak to end of the ECG complex. In the ST-segment, a moving-average-filter of length 10ms was used to further reduce the influence of high frequency noise.

The ratio between T-amplitude and QRS-amplitude (T/QRS ratio) was calculated from the filtered average ECG complex. To that end, the ECG baseline was determined based on the isoelectric period between P-wave and QRS-complex. Next, T-amplitude was calculated as the difference between T-peak and ECG baseline. Subsequently, QRS-amplitude was calculated as the maximum difference between R-peak and either Q- or S-peak. A T/QRS ratio was determined for every unipolar lead in all 5 head orientations in both dry and immersed conditions. These T/QRS values were used as outcome measure to compare the amplitudes of the ECG waveform between all conditions.

## Results

Tables 1 and 2 present all T/QRS ratios in the unipolar leads in dry and immersed condition, for all electrode positions and head orientations for experiment 1 and 2, respectively.

**Table 1:** T/QRS ratios in dry and immersed conditions (Experiment #1).

**Exp #1** **Dry**	**Head neutral**	**Ante-flexion**	**Rightward rotation**	**Leftward rotation**	**De-flexion**	**Maximal Δ**
**F0**	0.19	0.17	0.18	0.18	0.17	0.02
**Fz**	0.19	0.17	0.18	0.18	0.17	0.02
**O0**	0.19	0.17	0.17	0.18	0.17	0.02
**O1**	0.19	0.17	0.18	0.18	0.17	0.02
**O2**	0.19	0.17	0.17	0.18	0.17	0.02
**Maximal Δ**	0.00	0.00	0.01	0.00	0.00	
**Exp #1** **Immersed**	**Head neutral**	**Ante-flexion**	**Rightward rotation**	**Leftward rotation**	**De-flexion**	**Maximal Δ**
**F0**	0.14	0.12	0.16	0.13	0.12	0.04
**Fz**	0.16	0.17	0.16	0.16	0.12	0.05
**O0**	0.15	0.16	0.14	0.14	0.13	0.03
**O1**	0.13	0.16	0.13	0.14	0.11	0.05
**O2**	0.14	0.15	0.12	0.13	0.13	0.03
**Maximal Δ**	0.03	0.05	0.04	0.03	0.02	

T/QRS values are presented per unipolar scalp electrode position and head orientation. The maximal difference in T/QRS ratio between the electrode positions per head orientation, and between head orientation per electrode position is presented in the final columns and rows, respectively. Abbreviations: Exp #, experiment number.

**Table 2:** T/QRS ratios in dry and immersed conditions (Experiment #2).

**Exp #2** **Dry**	**Head neutral**	**Ante-flexion**	**Rightward rotation**	**Leftward rotation**	**De-flexion**	**Maximal Δ**
**F0**	0.18	0.17	0.19	0.17	0.17	0.02
**Fz**	0.17	0.17	0.19	0.17	0.17	0.02
**O0**	0.17	0.16	0.19	0.17	0.17	0.03
**O1**	0.17	0.16	0.19	0.17	0.17	0.03
**O2**	0.17	0.16	0.19	0.17	0.17	0.03
**Maximal Δ**	0.01	0.01	0.00	0.00	0.00	
*** ***	* *	* *	* *	* *	* *	
**Exp #2** **Immersed**	**Head neutral**	**Ante-flexion**	**Rightward rotation**	**Leftward rotation**	**De-flexion**	**Maximal Δ**
**F0**	0.13	0.14	0.12	0.12	0.11	0.03
**Fz**	0.13	0.12	0.13	0.11	0.07	0.06
**O0**	0.10	0.10	0.09	0.12	0.08	0.04
**O1**	0.09	0.09	0.08	0.11	0.06	0.05
**O2**	0.10	0.10	0.09	0.13	0.08	0.05
**Maximal Δ**	0.04	0.05	0.05	0.02	0.05	

T/QRS values are presented per unipolar scalp electrode position and head orientation. The maximal difference in T/QRS ratio between the electrode positions per head orientation, and between head orientation per electrode position is presented in the final columns and rows, respectively. Abbreviations: Exp #, experiment number.

**Fig 3:**
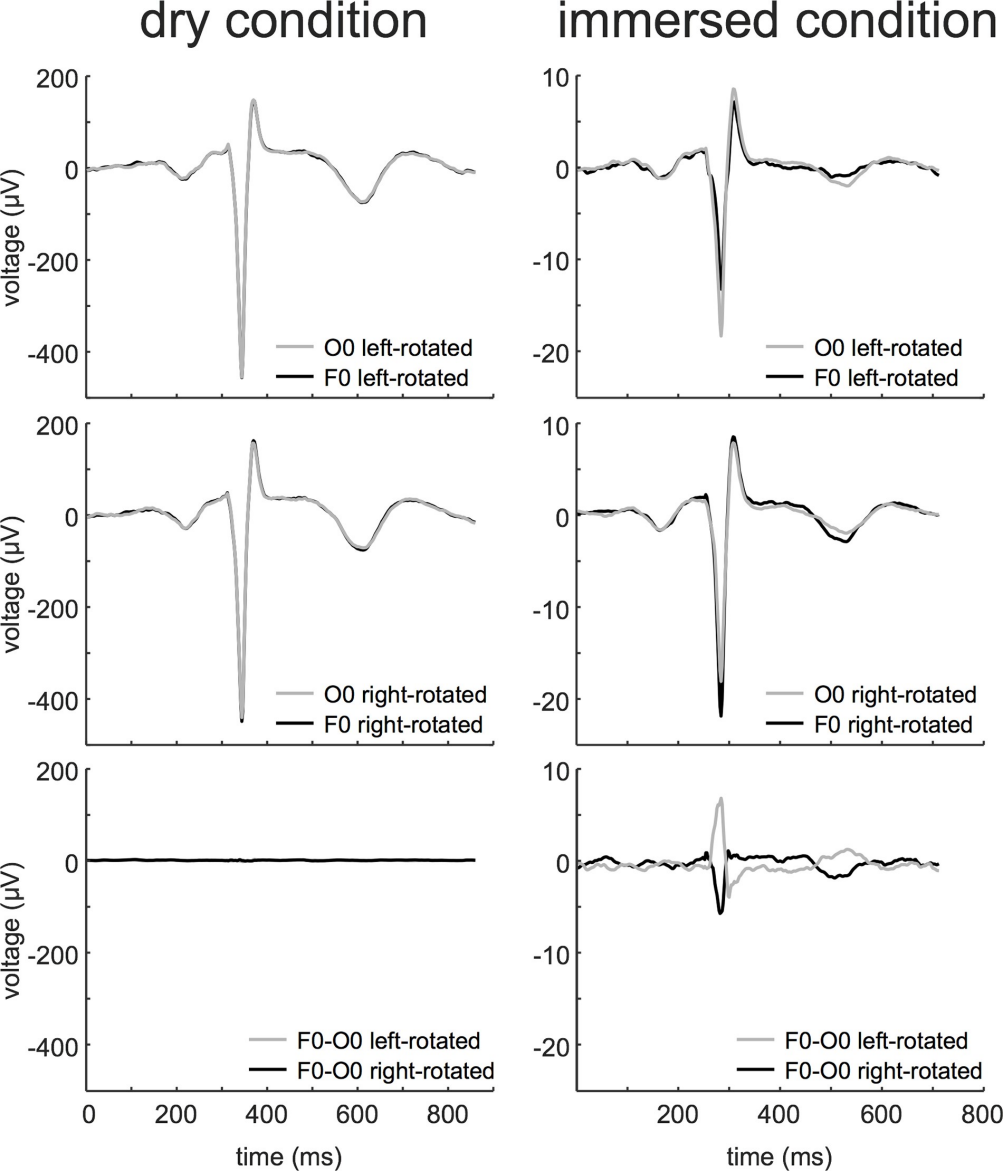
Examples of ECG complexes in dry and immersed condition. Unipolar (O0 and F0) and bipolar (F0-O0) scalp electrode signals with left- or right-rotated head during dry or immersed conditions.

During the dry condition we observed little to no difference in T/QRS ratio between different electrode positions (maximal difference between 0.00 and 0.01) in the unipolar leads. We observed minor differences in T/QRS ratio between head orientations (maximal difference between 0.02–0.03). During the immersed condition, the maximal T/QRS difference varied between 0.02–0.05 for electrode positions, and between 0.03–0.06 for head orientations, respectively. Fig 3 depicts examples of ECG complexes and T/QRS values in dry and immersed condition respectively (left panel versus right panel). The first and second row show contrasting electrode positions of *unipolar* leads O0 and F0. In dry condition, ECG waveform was similar in both leads, whether the head was left- or right-rotated. In contrast, in immersed condition the top row (head left-rotated) shows lower R and T peak amplitudes measured at the frontal lead (F0) compared to the occipital lead (O0). The middle row (head right-rotated) shows higher R and T peak amplitudes at the frontal compared to the occipital lead.

The bottom row of Fig 3 shows bipolar leads in both dry and immersed conditions. The left panel shows the bipolar leads in dry condition in left rotation and right rotation. No recognizable ECG signal could be estimated, as the electric potential differences between the electrodes were too small and noise-generated. In immersed condition however (right panel, third example), bipolar leads showed a recognizable ECG pattern, which differs in contrasting head orientations.

## Discussion

In this experimental study we examined the influence of electrode position and head orientation on the waveform of a single lead unipolar scalp ECG when recorded in a conductive medium. In dry condition ECG waveform was similar in all positions and orientations. In contrast, in immersed condition we observed ECG waveform differences between differently positioned electrodes at the scalp and between different head orientations.

We found that ECG waveform is independent of electrode position on the head in dry condition. The frontal and the occiput electrodes reflect the maximal possible difference between electrodes (F0-O0). Even these electrodes showed a similar ECG waveform in our experiment. In dry condition, cardiac currents are solely conducted through the body from the heart to the electrode at the scalp. As such, all electrode positions on the head reflect the same lead as the extension of the neck. In case head orientation towards the body changes, the path of the cardiac currents to the electrodes on the scalp remains equal. Hence, little to no influence was seen on the ECG waveform for different electrode positions (maximum variation was 0.01). We observed some minor variation between T/QRS value for different head orientations (maximum variation was 0.03). As different head orientations can only be measured consecutively and not simultaneously, factors changing over time, like lung air volume and distance to bath may influence ECG waveform. Additionally, flexing of the neck might yield some stress on the thorax and cause minor movement of the heart.

In immersed condition, we observed different ECG waveforms for the various electrode positions and head orientations. We showed that head rotations cause a change in absolute and relative T/QRS values. We also showed that the T/QRS baseline value differs between electrode locations, which may influence the amount of ST events in ST analysis. Vullings et al. showed that fetuses with a higher initial T/QRS baseline value have more ST events, irrespective of fetal condition.[8] The maximal difference in T/QRS between electrode positions was 0.05 and the maximal difference in T/QRS for different head orientations was 0.06. These differences in T/QRS values during immersed conditions can be explained because the electrical currents generated by the heart propagate to the scalp electrode both intracorporally and extracorporally (i.e., through the saline medium). The path of extracorporal conduction depends on the distance, position and presence of conductive media between the electrode and the heart, which varies for different electrode positions and head orientations. These differences in conduction path result in changes in the ECG amplitudes for each electrode and head orientation.

Besides larger changes in T/QRS values for immersed condition, we also observed lower absolute ECG amplitudes compared to the dry condition, as is seen from the amplitudes of the ECGs in Fig 3. In our experiment, we measured the ECG as a unipolar signal, where the difference between the scalp electrodes and the WCT is amplified. This difference is reduced during immersed conditions, due to the increased similarity of the scalp ECG and the WCT. It should be noted, however, that amplitudes of QRS-complex and T-wave were similarly suppressed. Therefore, we expect the effect of similarity between scalp electrode signal and WCT to be of little influence on T/QRS values.

Although we observed a more significant influence of electrode position and head orientation on the ECG waveform during immersed conditions for both experiment 1 and 2, results regarding changes in size of the T/QRS ratios were inconsistent. For example, in experiment 1, electrode O2 showed a difference of 0.01 in T/QRS between head oriented towards the right versus the left shoulder. On the contrary, experiment 2 showed a difference of 0.04 when the same head orientations were compared. These inconsistencies may be related to different conditions under which the experiments were conducted, such as lung air volume, electrode fixation, precise electrode positioning, magnitude of head movement, distance between electrode and bath tub, and water temperature. Although we attempted to minimize these effects, e.g. by only recording the ECG after complete exhalation and controlling water temperature, these conditions cannot be fully controlled. In future work, this experiment should be repeated multiple times in different individuals to enable more quantitative analysis of changes in ECG waveform.

Lindecrantz et al. compared fetal ECG morphology measured by bipolar and unipolar leads.[9] They found that, in case of bipolar measurements, the scalp ECG can invert due to the rotation of the fetal head during passage through the birth canal. We found comparable results in bipolar lead measurements in immersed condition. Altered electrode position and head orientation influence ECG waveform, as evidenced in Fig 3 where we show that under immersed conditions the ECG-waveform differs between contrasting head position (head rotated towards left or right shoulder, respectively). Because Lindecrantz et al. did not find any effect on ECG waveform due to head rotation in unipolar lead measurements, they suggested that this unipolar technique would be appropriate for fetal monitoring. We however now show that in immersed condition even unipolar lead signals are influenced by electrode position and head orientation.

This study has some limitations, which imply that extrapolating results to the fetal situation should be done with caution. The set up of this experiment is a simplified environment in comparison to the fetal situation and it does not include the effect of electrical currents from maternal tissues. We hypothesized that the fetal situation is comparable to the immersed condition. Besides, the sample size is limited to two experiments. To examine the effect of surrounding tissues on the waveform of the fetal scalp ECG in clinical practice would require comprehensive denotation or correction of the scalp electrode position and fetal head orientation during all stages of labor. This seems practically unfeasible. However, clinicians should be aware that fetal condition is not the only factor influencing the waveform of the fetal scalp ECG.

## Conclusions

In conclusion, in this experimental study we demonstrated that ECG waveforms recorded from unipolar as well as bipolar scalp leads are influenced by electrode position and head orientation when the subject is submerged in a conductive medium. This implies that intrapartum fetal monitoring based on ECG waveform obtained using scalp electrodes might be suboptimal, as there is no correction for fetal head neither orientation nor electrode position.

## Supporting information

S1 Results Digital average ECG complexes of experiment #1 for the 5 unipolar scalp electrodes in 6 positions under dry circumstances. (CSV)

S2 Results Digital average ECG complexes of experiment #1 for the 5 unipolar scalp electrodes in 6 positions while immersed. (CSV)

S3 Results Digital average ECG complexes of experiment #2 for the 5 unipolar scalp electrodes in 6 positions under dry circumstances. (CSV)

S4 Results Digital average ECG complexes of experiment #2 for the 5 unipolar scalp electrodes in 6 positions while immersed. (CSV)
